# Bilateral Inferior Head Turbinate Agenesis

**DOI:** 10.7759/cureus.71429

**Published:** 2024-10-14

**Authors:** Sacha Drabkin, Quentin Mat, Serge Daniel Le Bon

**Affiliations:** 1 Otolaryngology, Université Libre De Bruxelles, Érasme Campus, Brussels, BEL; 2 Otolaryngology, Centre Hospitalier Universitaire de Charleroi (CHU Charleroi), Charleroi, BEL; 3 Otolaryngology, Geneva University Hospitals, Geneva, CHE

**Keywords:** congenital nasal malformation, empty nose syndrome, nasal obstruction, trisomy 14, turbinate

## Abstract

Inferior nasal turbinates play a crucial role in conditioning inhaled air. While hypertrophy of these turbinates is a common cause of nasal obstruction in adults, congenital malformations are extremely rare. Only a few cases of unilateral agenesis have been reported. This report presents the first documented case of bilateral inferior head turbinate agenesis (BITHA).

A 22-year-old woman with a history of chronic nasal obstruction, sneezing, and nasal itching was diagnosed with BITHA. Her medical history included trisomy 14 mosaicism and other comorbidities. Clinical examination revealed mild facial dysmorphic features and agenesis of both inferior turbinate heads. Nasal endoscopy showed compensatory hypertrophy of the middle and posterior portions of the inferior turbinates. Following a one-month treatment with nasal corticosteroids and antihistamines, her nasal symptoms improved.

Trisomy 14 mosaicism is a rare genetic condition often associated with unspecific dysmorphic features. To our knowledge, BITHA has not been previously reported in such patients. This case suggests that BITHA could be a clinical feature for trisomy 14 mosaicism. Additionally, the absence of empty nose syndrome (ENS) in this patient, despite BITHA, provides insights into the potential iatrogenic origin of ENS.

BITHA is an exceptional congenital anomaly and may serve as a specific sign of trisomy 14 mosaicism. The lack of ENS in this case supports the hypothesis that ENS may arise from acquired rather than congenital anatomical changes.

## Introduction

Inferior nasal turbinates are involved in warming, humidifying, and filtering inhaled air [1]. Their hypertrophy is one of the most common causes of nasal obstruction in adults [2]. Nasal and sinus human anatomy is known to vary between individuals, such as agger nasi cells, nasal septum deviation, and concha bullosa [3]. However, malformations of inferior turbinates are extremely rare. Only a few cases of complete and unilateral agenesis of the inferior turbinates have been reported in the literature [4]. Here, to the best of our knowledge, we report the first case of bilateral inferior head turbinate agenesis (BIHTA).

## Case presentation

A 22-year-old woman presented to our clinic with a history of chronic nasal obstruction, anterior nasal discharge, sneezing, and nasal itching. Her past medical history included trisomy 14 mosaicism associated with interatrial septal aneurysm, asthma, overactive bladder, overweight, and adenoidectomy during childhood.

**Figure 1:**
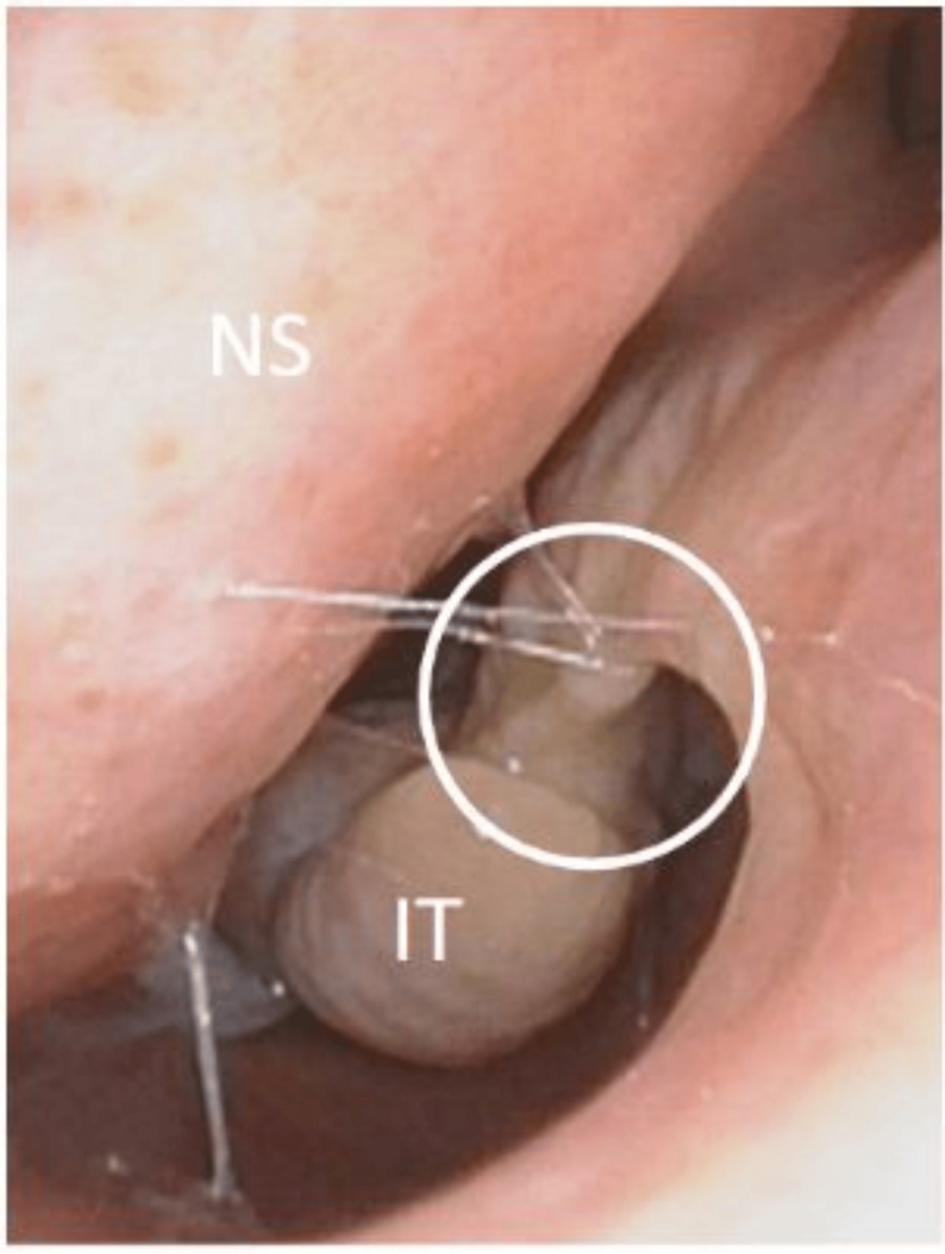
Endoscopic view of the left nasal cavity The circle shows the agenesis of the inferior turbinate. NS: nasal septum, IT: middle part of the inferior turbinate

**Figure 2:**
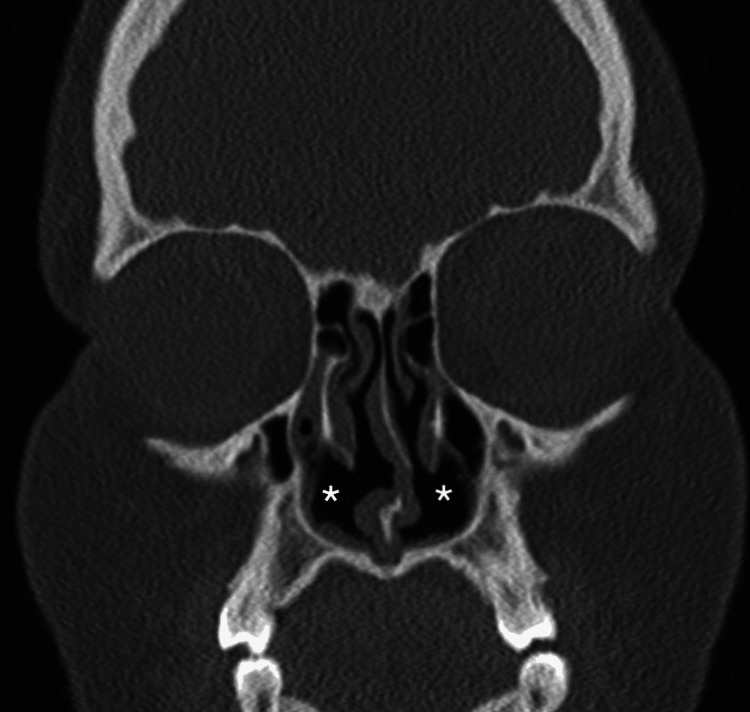
Coronal view CT scan of the sinus showing the bilateral agenesis of the head of the inferior turbinates The asterisk represents the agenesis of the head inferior turbinate. CT: computed tomography

**Figure 3:**
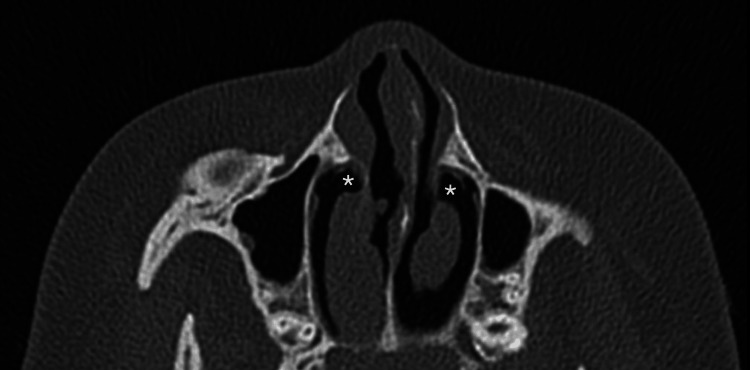
Axial view of the CT scan of the sinus showing the bilateral agenesis of the head of the inferior turbinates The asterisk represents the agenesis of the head inferior turbinate. CT: computed tompgraphy

Her clinical examination showed mild facial dysmorphic features with a depressed nasal bridge and a short nose. The agenesis of both inferior turbinate heads was observed during anterior rhinoscopy (Figure 1). Oral cavity and ear inspection were normal. Nasal endoscopy showed a compensatory hypertrophy of inferior turbinates in their middle and posterior portions as well as a clear nasal discharge. Skin prick tests were positive for cat and dog hairs that were present at home. A CT scan confirmed the diagnosis of agenesis of the anterior heads of the inferior turbinates and excluded sinusitis and other rhinological anomalies (Figures 2-3). One month of treatment with mometasone furoate nasal spray and oral H1-antihistamines helped relieve nasal obstruction and other nasal symptoms. At six-month follow-up, there was a significant improvement in symptoms.

## Discussion

Chromosome 14 is a rare and poorly described autosomal trisomy that is only compatible with human life in mosaic form [5]. They usually present unspecific dysmorphic features such as a broad nose, hypertelorism, ear abnormalities, micrognathia, and a cleft or highly arched palate [6]. For this reason, a correct diagnosis can take some time. Identifying more specific clinical signs could result in faster genetic assessment. To our knowledge, no case of BITHA has been previously reported, even in patients with trisomy 14 mosaicism. This clinical sign may not be systematically sought when this syndrome is suspected or confirmed. By reporting this case, we exhort physicians caring for patients with suspected trisomy 14 mosaicism to systematically look for BIHTA, as it may be a specific clinical sign.

In addition, the improvement in nasal breathing after initiation of anti-allergic treatment despite BIHTA is an interesting observation. Indeed, the empty nose syndrome (ENS) is a known but little-understood entity. This syndrome usually occurs after a turbinectomy (lower, medium, or both turbinates), which leads to a significant reduction in the head of the turbinate [7]. The exact pathophysiology is not yet defined. One of the theories presented concerns the “cooling” function of the nose involving trigeminal receptors [8]. As a result of the reduced mucous membrane volume, mucosal cooling would be compromised and contribute to the symptoms of ENS [9]. In our case, the patient did not have undergone turbinate surgery and did not have an ENS, although the mucous volume of the head of the inferior turbinate was markedly reduced. This could be an argument supporting the fact that an anatomical change occurring during life is necessary to cause ENS. On the other hand, our patient has a congenital malformation of the lower turbinates. Therefore, a modification of the distribution of the trigeminal receptors on the turbinates is not excluded.

## Conclusions

The case of BIHTA that we report here is an extremely rare congenital anomaly. To our knowledge, this is one of the first documented cases in the medical literature. This observation could represent a specific clinical sign of trisomy 14 mosaicism, a rare and poorly described genetic condition. It is important for clinicians suspecting or confirming a diagnosis of trisomy 14 mosaicism to include a thorough examination of the inferior turbinates in their assessment. Additionally, the improvement in nasal symptoms following anti-allergic treatment, despite BIHTA, sheds new light on the complexity of nasal syndromes such as empty nose syndrome.
